# James–Stein for the leading eigenvector

**DOI:** 10.1073/pnas.2207046120

**Published:** 2023-01-05

**Authors:** Lisa R. Goldberg, Alec N. Kercheval

**Affiliations:** ^a^Department of Economics and Consortium for Data Analytics in Risk, University of California, Berkeley, CA 94720; ^b^Aperio by BlackRock, Sausalito, CA 94965; ^c^Department of Mathematics, Florida State University, Tallahassee, FL 32306

**Keywords:** asymptotic regime, shrinkage, factor model, optimization, covariance matrix

## Abstract

Recent research identifies and corrects bias, such as excess dispersion, in the leading sample eigenvector of a factor-based covariance matrix estimated from a high-dimension low sample size (HL) data set. We show that eigenvector bias can have a substantial impact on variance-minimizing optimization in the HL regime, while bias in estimated eigenvalues may have little effect. We describe a data-driven eigenvector shrinkage estimator in the HL regime called “James–Stein for eigenvectors” (JSE) and its close relationship with the James–Stein (JS) estimator for a collection of averages. We show, both theoretically and with numerical experiments, that, for certain variance-minimizing problems of practical importance, efforts to correct eigenvalues have little value in comparison to the JSE correction of the leading eigenvector. When certain extra information is present, JSE is a consistent estimator of the leading eigenvector.

## Significance

Eigenvectors are used throughout the physical and social sciences to reduce the dimension of complex problems to manageable levels and to distinguish signal from noise. Our research identifies and mitigates bias in the leading eigenvector of a sample factor-based covariance matrix estimated in the high-dimension low sample size (HL) regime. The analysis illuminates how estimation error in a covariance matrix can affect quadratic optimization. Eigenvector estimation in the HL regime may be useful for disciplines, such as finance, machine learning, or genomics, in which high-dimensional variables need to be analyzed from a limited number of observations.

Averaging is the most important tool for distilling information from data. To name just two of countless examples, batting average is a standard measure of the likelihood that a baseball player will get on base, and an average of squared security returns is commonly used to estimate the variance of a portfolio of stocks.

The average can be the best estimator of a mean in the sense of having the smallest mean squared error. But a strange thing happens when considering a collection of many averages simultaneously. The aggregate sum of mean squared errors is no longer minimized by the collection of averages. Instead, the error can be reduced by shrinking the averages toward a common target, even if, paradoxically, there is no underlying relation among the quantities.

For baseball players, since an individual batting average incorporates both the true mean and estimation error from sampling, the largest observed batting average is prone to be overestimated and the smallest underestimated. That is why the aggregate mean squared error is reduced when the collection of observed averages are all moved toward their center.

This line of thinking has been available at least since Sir Francis Galton introduced “regression towards mediocrity” in 1886. Still, Charles Stein surprised the community of statisticians with a sequence of papers about this phenomenon beginning in the 1950s. Stein showed that it is always possible to lower the aggregate squared error of a collection of three or more averages by explicitly shrinking them toward their collective average. In 1961, Stein improved and simplified the analysis in collaboration with Willard James. The resulting empirical James–Stein shrinkage estimator (JS) launched a new era of statistics.

This article describes “James–Stein for eigenvectors” (JSE), a recently discovered shrinkage estimator for the leading eigenvector of an unknown covariance matrix. A leading eigenvector is a direction in a multidimensional data set that maximizes explained variance. The variance explained by the leading eigenvector is the leading eigenvalue.

Like a collection of averages, a sample eigenvector is a collection of values that may be overly dispersed. This can happen in the high-dimension low sample size (HL) regime when the number of variables is much greater than the number of observations. In this situation, the JSE estimator reduces excess dispersion in the entries of the leading sample eigenvector. The HL regime arises when a relatively small number of observations are used to explain or predict complex high-dimensional phenomena, and it falls outside the realm of classical statistics. Examples of such settings include genome-wide association studies (GWAS), such as (1) and (2), in which characteristics of a relatively small number of individuals might be explained by millions of single nucleotide polymorphisms (SNPs); machine learning in domains with a limited number of high-dimensional observations, such as in (3); and finance, in which the number of assets in a portfolio can greatly exceed the number of useful observations.

We work in the context of factor models and principal component analysis, which are used throughout the physical and social sciences to reduce dimension and identify

the most important drivers of complex outcomes. Principal component analysis (PCA) is a statistical technique that uses eigenvectors as factors. The results in this article are set in the context of a one-factor model that generates a covariance matrix with a single spike. This means that the leading eigenvalue is substantially larger than the others. We do not provide a recipe for practitioners working in higher-rank contexts; our goal is to describe these ideas in a setting in which we can report the current state of the theory. However, similar results are reported experimentally for multifactor models by Goldberg et al. (4), and continuing theoretical work indicates that the success of this approach is not limited to the one-factor case.

We begin this article by describing the JS and JSE shrinkage estimators side by side, in order to highlight their close relationship. We then describe three asymptotic regimes, low-dimension high sample size (LH), high-dimension high sample size (HH), and high-dimension low sample size (HL), in order to clarify the relationship between our work and the literature. Subsequently, we describe an optimization-based context in which a high-dimensional covariance matrix estimated with the JSE estimator performs substantially better than eigenvalue correction estimators coming from the HH literature. We describe both theoretical and numerical supporting results for performance metrics relevant to minimum variance optimization.

This article focuses on high-dimensional covariance matrix estimation via shrinkage of eigenvectors, rather than eigenvalues or the entire covariance matrix. It relies on results from the HL regime and emphasizes optimization-based performance metrics. The bulk of the existing high-dimensional covariance estimation literature concerns correction of biased eigenvalues or provides results only in the HH regime or focuses on metrics that do not take account of the use of covariance matrices in optimization.

## James–Stein for Averages

Suppose there are *p* >  3 unknown means *μ* = (*μ*_1_, *μ*_2_, …, *μ*_*p*_) to be estimated. We observe a fixed number of samples and compute the corresponding sample averages *z* = (*z*_1_, *z*_2_, …, *z*_*p*_).

It is common practice to use *z*_*i*_ as an estimate for the unobserved mean value *μ*_*i*_, and this may be the best one can do if only a single mean is estimated. The discovery of Stein (5) and James and Stein (6) is that a better estimate is obtained by shrinking the sample averages toward their collective average.

Let $m\left(z\right)=\sum_{i=1}^{p}z_{i}/p$ denote the collective average, and **1** = (1, 1, …, 1), the *p*-dimensional vector of 1s. With certain normality assumptions, James and Stein define:

[1] $$\begin{array}{c} {\hat{\mu }}^{\mathrm{JS}}=m\left(z\right)1+c^{\;\mathrm{JS}}\left(z-m\left(z\right)1\right). \end{array}$$

The shrinkage constant *c*^ JS^ is given by

[2] $$\begin{array}{c} c^{\;\mathrm{JS}}=1-\frac{\nu^{2}}{s^{2}\left(z\right)}, \end{array}$$

where

[3] $$\begin{array}{c} s^{2}\left(z\right)=\frac{1}{p-3}\sum_{i=1}^{p}{\left(z_{i}-m\left(z\right)\right)}^{2} \end{array}$$

is a measure of the variation of the sample averages *z*_*i*_ around their collective average *m*(*z*), and *ν*^2^ is an estimate of the conditional variance of each sample average around its unknown mean. The value of *ν*^2^, a measure of the noise affecting each observed average, must be either assumed or estimated independently of *s*^ 2^(*z*), and is sometimes tacitly taken to be 1.

The observable quantity *s*^ 2^(*z*) incorporates both the unobserved variation of the means and the noise *ν*^2^. The term *ν*^2^/*s*^2^(*z*) in Eq. **2** can be thought of as an estimated ratio of noise to the sum of signal and noise. **Eq. 1** calls for a lot of shrinkage when the noise dominates the variation of the sample averages around their collective average and only a little shrinkage when the reverse is true. Readers may consult Efron and Morris (7, 8), and Efron (9) for more complete discussion and motivation behind formula [**1**] as an empirical Bayes estimator.

James and Stein showed that the JS estimator ${\hat{\mu }}^{\;\text{JS}}$ is superior to *z* in the sense of expected mean squared error,

[4] $$\begin{array}{c} E_{\mu ,\nu }\left[|,{\hat{\mu }}^{\mathrm{JS}},{-\mu |}^{2}\right]<E_{\mu ,\nu }\left[{\left|z-\mu \right|}^{2}\right]. \end{array}$$

For any fixed *μ* and *ν*, the conditional expected mean squared error is improved when using ${\hat{\mu }}^{\;\text{JS}}$ instead of *z*. This result comes with an unavoidable caveat: *z* remains the optimal estimate when *p* = 1 and *p* = 2 and sometimes when *p* = 3.

Suppose we have *p* >  3 baseball players, and, for *i* = 1, 2, …, *p*, player *i* has true batting average *μ*_*i*_, meaning that in any at-bat, the player has a probability *μ*_*i*_ of getting a hit. This probability is not observable, but we do observe, say over the first 50 at-bats of the season, the realized proportion *z*_*i*_ of hits. Assuming we know *ν*^2^ or have an independent way to estimate it, Eq. **1** improves on the *z*_*i*_ as estimates of the true means *μ*_*i*_.

This example lends intuition to the role of the noise to signal-plus-noise ratio *ν*^2^/*s*^ 2^(*z*) in the JS shrinkage constant. If the true batting averages differ widely, but the sample averages tend to be close to the true values, then Eq. **1** calls for little shrinkage, as appropriate. Alternatively, if the true averages are close together, but the sampling error is large, a lot of shrinkage makes sense. The JS estimator properly quantifies the shrinkage and interpolates between these extremes.

## James–Stein for Eigenvectors

Consider a sequence of *n* independent observations of a variable of dimension *p*, drawn from a population with unknown covariance matrix *Σ*. The *p* × *p* sample covariance matrix *S* has the spectral decomposition:

[5] $$\begin{array}{c} S=\lambda^{2}hh^{⊤}+\lambda_{2}^{2}v_{2}v_{\;2}^{⊤}+\lambda_{3}^{2}v_{\;3}v_{3}^{⊤}\cdots +\lambda_{p}^{2}v_{p}v_{p}^{⊤}. \end{array}$$

in terms of the nonnegative eigenvalues *λ*^2^ ≥ *λ*_2_^2^ ≥ ⋯ ≥ *λ*_*p*_^2^ ≥ 0 and orthonormal eigenvectors {*h*, *v*_2_, …, *v*_*p*_} of *S*. Our interest is primarily in the leading eigenvalue *λ*^2^ and its corresponding eigenvector *h* when *p* >  > *n*. In what follows, the sample eigenvector *h* plays the role of the collection of sample averages *z* in the previous discussion.

In classical statistics with fixed *p*, the sample eigenvalues and eigenvectors are consistent estimators of their population counterparts when the population eigenvalues are distinct. This means that the sample estimates converge to the population values as *n* tends to infinity. However, this may fail when the dimension tends to infinity. The purpose of JSE is to provide an empirical estimator improving on the sample eigenvector *h* in the HL setting.

JSE is a shrinkage estimator, analogous to JS, that improves on *h* by having a lower squared error with high probability and leading to better estimates of covariance matrices for use in quadratic optimization. Goldberg, Papanicolaou, and Shkolnik introduced and analyzed the JSE estimator in (10) as a means to improve the output of quadratic optimization. It is further developed and extended by Goldberg et al. (4) and Gurdogan and Kercheval (11). The connection between JSE and JS first appears in Shkolnik (12) in the context of a single spiked covariance model.

The JSE estimator *h*^ JSE^ is defined by shrinking the entries of *h* toward their average *m*(*h*), just as in Eq. **1**:

[6] $$\begin{array}{c} h^{\;\mathrm{JSE}}=m\left(h\right)1+c^{\;\mathrm{JSE}}\left(h-m\left(h\right)1\right), \end{array}$$

where the shrinkage constant *c*^ JSE^ is

[7] $$\begin{array}{c} c^{\;\mathrm{JSE}}=1-\frac{\nu^{2}}{s^{2}\left(h\right)}, \end{array}$$

where

[8] $$\begin{array}{c} s^{2}\left(h\right)=\frac{1}{p}\sum_{i=1}^{p}{\left(\lambda ,h_{i},-,\lambda ,m,\left(h\right)\right)}^{2} \end{array}$$

is a measure of the variation of the entries of *λh* around their average *λm*(*h*), and *ν*^2^ is equal to the average of the nonzero smaller eigenvalues of *S*, scaled by 1/*p*,

[9] $$\begin{array}{c} \nu^{2}=\frac{tr\left(S\right)-\lambda^{2}}{p\cdot (n-1)}. \end{array}$$

As with JS, JSE calls for a lot of shrinkage when the average of the nonzero smaller eigenvalues dominates the variation of the entries of *λh* around their average and only a little shrinkage when the reverse is true. The estimator *h*^ JSE^ improves on the sample leading eigenvector *h* of *S*, as we describe below, by reducing its angular distance to the population eigenvector.

To state a precise result, we introduce the factor model framework in which we are applying JSE, as initiated in (10) and elaborated in (11). Factor models are widely used to reduce dimension in settings where there are a relatively small number of drivers of a complex outcome. The prototype is a one-factor model:

[10] $$\begin{array}{c} r=\beta f+\epsilon , \end{array}$$

where *r* is a *p*-vector that is the sole observable, *β* is a *p*-vector of factor loadings, the scalar *f* is a common factor through which the observable variables are correlated, and *ϵ* is a *p*-vector of variable-specific effects that are not necessarily small but are homogeneous and uncorrelated with *f* and each other. Setting the factor variance to be *σ*^2^ and the specific variance to be *δ*^2^, the population covariance matrix takes the form:

[11] $$\begin{array}{c} \Sigma =\sigma^{2}\beta \beta^{⊤}+\delta^{2}I, \end{array}$$

and *β* is its leading eigenvector.

Our theoretical results are asymptotic in the number of variables *p*, so we introduce a fixed sequence of scalars {*β*_*i*_}_*i* = 1_^∞^, from which we draw factor loadings. Suppressing dependence on dimension in our notation, let *β* be the *p*-vector whose entries are the first *p* elements of the fixed sequence. To prevent asymptotic degeneracy of the *p*-indexed sequence of models, we impose the normalizing condition that ${\left|\beta \right|}^{2}/p=\left(1/p\right)\sum_{i=1}^{p}\beta_{i}^{2}$ tends to a finite positive limit as *p* → ∞.

Any nonzero multiple of an eigenvector is an eigenvector, so we define the distance between population and estimated eigenvectors as the smallest positive angle, denoted ∠, between representatives.

Theorem 1 (10). Assume that the angle ∠(*β*, **1**) tends to a limit strictly between zero and *π*/2.

Then, in the limit as *p* → ∞ with *n* fixed,

[12] $$\begin{array}{c} ∠\left(h^{\;\mathrm{JSE}},\beta \right)<∠\left(h,\beta \right) \end{array}$$

almost surely.

The proofs in (10) assume the equivalent hypotheses that the mean *m*(*β*) and dispersion *d*(*β*) have finite positive limits, where

[13] $$\begin{array}{c} d^{2}\left(\beta \right)=\frac{1}{p}\sum_{i=1}^{p}{\left(\frac{\beta_{i}-m\left(\beta \right)}{m(\beta )}\right)}^{2}. \end{array}$$

A limiting mean of zero corresponds to a limiting angle between *β* and **1** of *π*/2, in which case *h*^ JSE^ reduces to *h* and the strict inequality of *Theorem 1* becomes a weak inequality.

**Fig. 1.**
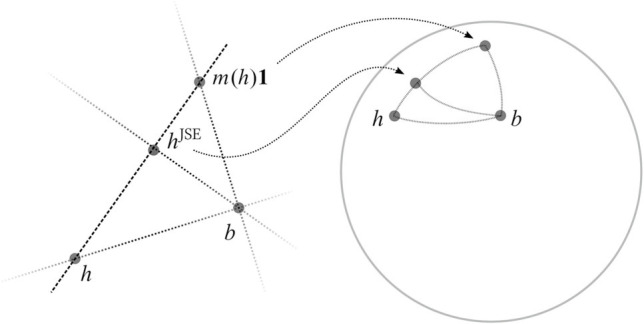
Shrinkage of the sample eigenvector *h* along the line connecting *h* and *m*(*h*)**1** in Euclidean space (*Left*) and projected on the unit sphere (*right*).

The unit eigenvector *b* = *β*/|*β*| is featured in our illustration of [**12**] in Fig. 1. The left panel shows JSE shrinkage as defined by Eq. **6**. The right panel shows an equivalent formulation of JSE shrinkage in terms of vectors on the unit sphere obtained by normalization.

The conclusion of *Theorem 1* is equivalent to the statement that the JSE estimator reduces the Euclidean distance between normalized representatives

[14] $$\begin{array}{c} \left|\frac{h^{\;\mathrm{JSE}}}{|h^{\;\mathrm{JSE}}|},-,b\right|<\left|h-b\right|, \end{array}$$

when they are chosen to lie in the same hemisphere. This is due to the elementary relation (1/2)|*u* − *v*|^2^ = 1 − cos∠(*u*, *v*) for any unit vectors *u*, *v*.

*Theorem 1* guarantees that the angle between *h*^ JSE^ and *b* becomes smaller than the angle between *h* and *b* for *p* sufficiently large as long as ∠(*β*, **1**) tends to a value in the interval (0, *π*/2) and |*β*|^2^/*p* tends to a positive value as *p* tends to infinity. We explore the magnitude of improvement offered by JSE on a data set of *n* = 40 observations and *p* = 50, 100, 200, and 500 variables. Gaussian data are simulated with the factor model Eq. **10**, with *σ* = 0.16, *δ* = 0.60, and *β* generated by applying an appropriate affine transformation to pseudorandom draws from a normal distribution so that |*β*|^2^/*p* = 1 and ∠(*β*, **1**) is as desired. The choice of these parameters is motivated by equity markets, as described in (4). We consider small, medium, and large angles, ∠(*β*, **1**)=0.174, 0.785, and1.40 radians, equivalently, 10, 45, and 80°. For each fixed *p* and *β*, our experiment relies on 1,000 simulated paths.

Table 1 shows the mean and median difference,

[15] $$\begin{array}{c} D=∠\left(h,b\right)-∠\left(h^{\;\mathrm{JSE}},b\right), \end{array}$$

**Table 1.** Improvement (D) measured in radians, by JSE over the sample eigenvector as an estimator of the population eigenvector.

∠(β,1)				Interquartile	
(Radians)	p	Mean D	Median D	Range D	P(D > 0)
0.174	50	0.276	0.289	0.051	0.996
	100	0.316	0.345	0.055	0.989
	200	0.276	0.367	0.066	0.967
	500	0.328	0.370	0.071	0.983
0.785	50	0.066	0.066	0.025	0.995
	100	0.069	0.069	0.028	0.998
	200	0.070	0.068	0.027	1.000
	500	0.069	0.067	0.025	1.000
1.396	50	0.000	0.002	0.004	0.724
	100	0.001	0.003	0.004	0.762
	200	0.002	0.003	0.004	0.757
	500	0.002	0.002	0.003	0.867

Gaussian data are generated from the factor model [10] with *σ* = 0.16, *δ* = 0.60, and |*β*|^2^/*p* = 1. Results are based on 1,000 simulations of *n* = 40 observations for each value of ∠(β,1) and *p*. Average and median improvement are uniformly positive, and they increase as ∠(β,1) decreases. Results are consistent across values of *p* considered.

along with its interquartile range and the probability that *D* is positive. The mean, median, and interquartile range of improvement *D* by JSE are small and positive for the largest angle we consider, ∠(*β*, **1**)=1.40 radians, close to a right angle, and increase materially as that angle diminishes. The probability that *D* is positive exceeds 0.72 in all cases and exceeds 0.96 for the two smaller angles. The results are stable across values of *p*, consistent with the hypothesis that *n* = 40 and *p* = 50 are effectively in the asymptotic regime for the factor model that we specified.

### A More General Shrinkage Target.

In Eqs. **1** and **6**, JS and JSE reduce excess dispersion in an estimated vector of interest relative to a shrinkage target, *τ* = *m*(⋅)**1**, with constant entries. Efron and Morris (7) describe the JS estimator for a more general shrinkage target, where the dispersionless vector *m*(⋅)**1** is replaced by an initial guess *τ* ∈ *R*^*p*^ for the unknown *μ*. In that case, the JS estimator becomes

[16] $$\begin{array}{c} {\hat{\mu }}^{\mathrm{JS}}=\tau +c^{\;\mathrm{JS}}\left(z-\tau \right), \end{array}$$

where *c*^ JS^ is defined relative to *τ*, with

[17] $$\begin{array}{c} s^{2}\left(h\right)=\sum_{i=1}^{p}{\left(z_{i}-\tau_{i}\right)}^{2}/\left(p-2\right). \end{array}$$

We describe a similar generalization of *Theorem 1*. As we did for factor loadings *β*, we introduce a fixed sequence of scalars {*τ*_*i*_}_*i* = 1_^∞^, from which we draw coordinates of a shrinkage target vector *τ*. In the previous case, *τ*_*i*_ = 1 for all *i*. Continuing to suppress dimension in our notation, let *τ* be the *p*-vector whose entries are the first *p* elements of the sequence. To avoid degeneracy, we again impose the normalizing assumption that |*τ*|^2^/*p* tends to a finite positive limit as *p* → ∞.

For any *p*-vector *y*, denote the the orthogonal projection of *y* onto *τ* by

[18] $$\begin{array}{c} P_{\tau }\left(y\right)=\left\langle y,\tau \right\rangle \frac{\tau }{{\left|\tau \right|}^{2}}. \end{array}$$

Define the generalized variance relative to *τ* as

[19] $$\begin{array}{cc} v_{\tau }^{2}\left(y\right) & =\frac{1}{p}{\left|y-P_{\tau }\left(y\right)\right|}^{2}, \end{array}$$

and define the generalized shrinkage constant

[20] $$\begin{array}{c} c_{\tau }^{\;\mathrm{JSE}}=1-\frac{\nu^{2}}{\lambda^{2}v_{\tau }^{2}\left(h\right)}, \end{array}$$

where *ν*^2^ is defined as before and we assume *h* ≠ *P*_*τ*_(*h*). We may now define the generalized JSE estimator as

[21] $$\begin{array}{c} h_{\tau }^{\mathrm{JSE}}=P_{\tau }\left(h\right)+c_{\tau }^{\;\mathrm{JSE}}\left(h-P_{\tau }\left(h\right)\right), \end{array}$$

which depends only on the line determined by *τ*​.

Theorem 2 (10). Assume that the angle ∠(*β*, *τ*) tends to a limit strictly between zero and *π*/2.

Then, in the limit as *p* → ∞ with *n* fixed,

[22] $$\begin{array}{c} ∠\left(h_{\tau }^{\;\mathrm{JSE}},\beta \right)<∠\left(h,\beta \right) \end{array}$$

almost surely.

The proof of *Theorem 2* is a formal generalization of the proof of theorem 3.1 in (10), with the original target **1** replaced by *τ*, as long as the nondegeneracy condition on |*τ*|^2^/*p* is satisfied. When the entries of *τ* are all ones, we recover *Theorem 1* as a special case of *Theorem 2*.

The analogy of JSE with JS suggests viewing *τ* as a guess at the identity of the true eigenvector *β*. An alternative is to think of *τ* as an exogenously imposed constraint in a variance-minimizing optimization. In this situation, JSE corrects the sample eigenvector in the direction of *τ* to reduce optimization error. The effectiveness of this correction is controlled by the angle between *β* and *τ*, ∠(*β*, *τ*) as well as |*β*^2^|/*p* and |*τ*|^2^/*p*. This alternative perspective allows us to think of a *τ*-indexed family of biases in the sample eigenvector *h*.

### A Consistent Estimator.

An extension of the generalized JSE estimator developed by Gurdogan and Kercheval in (11) incorporates multiple targets to further reduce estimation error. The result depends on a specific collection of *k* = *k*(*p*)< *p* linearly independent target vectors {*τ*^1^, *τ*^2^, …, *τ*^*k*^}. Letting *τ* denote the (*p* × *k*)-dimensional matrix whose columns are the *τ*^*i*^s, the orthogonal projection of a *p*-vector *y* onto the *k*-dimensional space spanned by the columns of *τ* is

[23] $$\begin{array}{c} P_{\tau }\left(y\right)=\tau {\left(\tau^{⊤}\tau \right)}^{-1}\tau^{⊤}y. \end{array}$$

Suppose we know the rank ordering of the betas *β*_1_, *β*_2_, …, *β*_*p*_, but not their actual values. Group the betas into *k* ordered quantiles, where *k* is approximately $\sqrt{p}$. For *i* = 1, 2…,*k*, define the target vector *τ*^*i*^ = (*a*_1_, *a*_2_, …, *a*_*p*_), where *a*_*j*_ = 1 if *β*_*j*_ belongs to group *i*, and zero otherwise.

Theorem 3 (11). Let the number *n* of observations be fixed. For *τ* equal to the (*p* × *k*)-dimensional matrix whose columns are the *τ*^*i*^s defined from the rank ordering of betas as above, the JSE estimator defined by Eq. **21** is a consistent estimator of *b* in the sense that

[24] $$\begin{array}{c} \lim_{p\to \infty }∠\left(h_{\tau }^{\;\mathrm{JSE}},b\right)=0 \end{array}$$

almost surely.

In (11), it is shown that the full rank ordering is not needed; only the ordered groupings are used.

## Three Regimes

The two James–Stein estimators, for averages and for the leading eigenvector, are structurally parallel, but the current state of theory guarantees their performance in different settings. The dominance of JS over the sample mean expressed in inequality 4 holds in expectation, typically under normality assumptions, for finite *p* >  3. In contrast, the JSE theory of *Theorems 1* and *3* is asymptotic in the HL regime and is nonparametric, courtesy of the strong law of large numbers.

The relevance of the HL regime to the analysis of scientific data was recognized as early as 2005, by Hall et al. (13). The 2018 article by Aoshima et al. (14) surveys results on the HL regime.

The HL regime stands in contrast to the low-dimension high sample size (LH) regime of classical statistics, where the number of variables *p* is fixed and the number of observations *n* tends to infinity. In the LH regime, a sample covariance matrix based on identically distributed, independent observations is a consistent estimator of the population covariance matrix, converging in expectation as *n* tends to infinity. Different effects emerge in the high-dimension high sample size (HH) regime, in which both *p* and *n* tend to infinity. The HH regime is part of random matrix theory, dating back to the 1967 work of Marčenko and Pastur (15). This three-regime classification of data analysis is discussed by Jung and Marron in their 2009 article (16).

Placing any particular finite problem into an asymptotic context, whether LH, HL, HH, or something in between, requires specifying how the model is to be extended asymptotically. For LH, this means letting the number of independent observations grow, but the HH and HL regimes require defining a sequence of models of increasing dimension. This extension was natural in early works from random matrix theory that characterized the limiting spectra of standard Gaussian variables in the HH regime. Johnstone (17) looks at the HH spectrum of eigenvalues in a spiked model, where the eigenvalues of a fixed-dimensional set of eigenvectors are substantially larger than the remaining eigenvalues. The covariance matrix corresponding to the factor model, Eq. **10** is spiked. In some settings, it can be beneficial to estimate the spiked covariance model guided by *Theorems 1* and *3* from the HL regime.

**Fig. 2.**
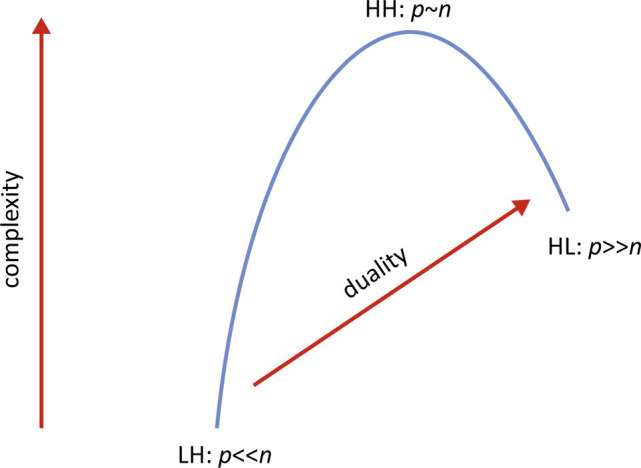
Three asymptotic regimes for data analysis. LH is the low-dimension high sample size regime of classical statistics. HH is the high-dimension high sample size regime of classical random matrix theory. HL is the high-dimension low sample size regime of alternative random matrix theory. HH tends to be more complex than HL because duality arguments allow some features of classical statistics to emerge in the HL regime.

A schematic diagram of the three regimes is in Fig. 2. Duality enables us to use classical statistics to obtain results in the HL regime. This has been observed by various researchers, including Shen et al. (18) and Wang and Fan (19) and used in (10). For example, if *Y* is our *p* × *n* data matrix with *p* >  *n*, the *p* × *p* sample covariance matrix *YY*^⊤^/*n* has rank at most *n*. If we consider the *n* × *n* dual matrix *S*^*D*^ = *Y*^⊤^*Y*/*p*, it has a fixed dimension in the HL regime. The nonzero eigenvalues of *S*^*D*^ and *S* are related by the multiplicative factor *p*/*n*, and the eigenvectors are related by left multiplication by *Y* or *Y*^⊤^. Since, for *S*^*D*^, the roles of *p* and *n* are reversed, methods from classical statistics apply.

## High-Dimensional Covariance Matrix Estimation

Eigenvalue adjustment to improve covariance performance metrics, or loss functions, goes back at least to Stein’s 1956 and 1986 articles (20) and (21). In this section, we discuss aspects of the literature.

In their 2018 article (22), Donoho, Gavish, and Johnstone emphasize the dependence of the optimal estimator on the choice of performance metric. Like Stein (21), they consider estimators obtained by varying the eigenvalues while keeping the sample eigenvectors fixed. In describing an oracle estimator for their spiked covariance model in the HH regime, they write:

The oracle procedure does not attain zero loss since it is “doomed” to use the eigenbasis of the empirical covariance, which is a random basis corrupted by noise, to estimate the population covariance.

This situation is reasonable in the context they consider in which there is no prior information, other than data, about the eigenvectors. As indicated in (11), prior information can allow for the correction of a wide range of eigenvector biases in the HL regime.

Similar themes emerge from a series of articles (23–28), by Ledoit and Wolf. Beginning in 2003, these papers explore high-dimensional covariance matrix estimation with applications to financial portfolio construction and other disciplines. As in the paper by Donoho et al. (22), Ledoit and Wolf (28), consider “the class of rotation-equivariant estimators”.

Ledoit and Wolf write:

Rotation equivariance is appropriate in the general case where the statistician has no a priori information about the orientation of the eigenvectors of the covariance matrix…
The fact that we keep the sample eigenvectors does not mean that we assume they are close to the population eigenvectors. It only means that we do not know how to improve upon them.

In earlier papers, Ledoit and Wolf consider estimators that shrink a sample covariance matrix toward a target. Some of these estimators modify the sample eigenvectors. By implementing a spiked shrinkage target in (25), Ledoit and Wolf provide prior structural information to the estimator. For the JSE estimator, that structural information is in the form of a factor model and the positive mean assumption on the leading population eigenvector.

In their 2017 article, Wang and Fan (19) develop the S-POET eigenvalue shrinkage estimator, which can be applied to the spiked covariance model in the HH and certain HL regimes. They evaluate S-POET with performance metrics based on the relative spectral norm, the relative Frobenius norm, the spectral norm, and the max norm. Their candidate estimators, again, use the sample eigenvectors. In the absence of structural information, they also remark that “correction for the biases of estimating eigenvectors is almost impossible.”

Despite the challenges of characterizing or correcting sample eigenvectors in high dimensions, there are streams of literature on the subject in both the HH and HL regimes. Some of the literature concerns consistency of sample eigenvectors under different modeling assumptions. HH references include Paul (29), Nadler (30), Mestre (31), and Johnstone and Lu (32). A 2018 survey by Johnstone and Paul (33) has an extensive reference list. HH results that are partial analogs of our findings include Montanari (34) and Montanari and Venkataramanan (35), who study estimation of singular vectors for low-rank matrices using approximate message passing (AMP) algorithms. In a 2022 article (36), Zhong, Su, and Fan describe a Bayes AMP algorithm to estimate principal components in the HH regime. Techniques from the HH regime have been applied to improve optimized portfolios; see, for example, the 2012 paper by Menchero and Orr (37), and the 2013 publication by El Karoui (38).

For the HL regime, asymptotics and estimation of eigenvectors have been studied in work previously cited and, among others, Ahn et al. (39), Jung et al. (40), Lee et al. (41), and Jung (42).

In the next section, we introduce a focus on optimization error and relevant performance metrics. We show that JSE eigenvector shrinkage, perhaps surprisingly, can substantially dominate the gains due to eigenvalue correction in optimization-based performance metrics.

## JSE Corrects an Optimization Bias

Estimated covariance matrices are used in quadratic optimization, which chooses coefficients to minimize the variance of a linear combination of random variables subject to constraints. In what follows, we evaluate estimators of high-dimensional spiked covariance matrices with performance metrics that measure the accuracy of optimized quantities.

We present simulations of practical situations where JSE materially improves optimization-based performance metrics while eigenvalue corrections can have little effect. Our simulations illustrate results from (10) and (11) showing the dependence of optimization-based performance metrics on the optimization bias as the number of variables *p* tends to infinity and the lack of dependence of these metrics on errors in eigenvalues. Our context and examples are taken from financial economics but our results apply in any discipline where spiked covariance models are used as inputs to quadratic optimization.

### Quantitative Portfolio Construction.

From a universe of *p* financial securities, there are countless ways to construct a portfolio. We focus on quantitative portfolio construction, which has relied on mean–variance optimization since Markowitz (43). In this framework, a portfolio is represented by a vector whose *i*th entry is the fraction or weight of the portfolio invested in security *i*. A portfolio is efficient if it has minimum forecast variance subject to constraints, and the search for efficient portfolios is central to quantitative finance. The simplest efficient portfolio is minimum variance.

A fully invested but otherwise unconstrained minimum variance portfolio is the solution ${\hat{w}}^{*}$ to the mean-variance optimization problem

[25] $$\begin{array}{c} \begin{array}{cc} & \min_{w\in R^{p}}\;w^{⊤}\hat{\Sigma }w\\ & \;\text{subject to:}\\ & w^{⊤}1=1, \end{array} \end{array}$$

where the *p* × *p* matrix $\hat{\Sigma }$ is a nonsingular estimate of the unknown true security covariance matrix *Σ*. If the estimate $\hat{\Sigma }$ is derived from observed data, then ${\hat{w}}^{*}$ is a data-driven approximation of the true optimum w^*^, defined as the solution to [**25**] with $\hat{\Sigma }$ replaced by *Σ*.

### Performance Metrics and Optimization.

We review three performance metrics that are sensitive to different aspects of the impact of covariance matrix estimation error on optimization.

The variance forecast ratio (VFR) is the quotient of estimated by true variance of a linear combination of random variables. Considered in 1956 by Stein (20) for arbitrary combinations, the VFR can be substantially less than the maximum value 1 when it is applied to an optimized quantity like a minimum variance portfolio:

[26] $$\begin{array}{c} \mathrm{VFR}\left({\hat{w}}^{*}\right)=\frac{{\hat{w^{*}}}^{⊤}\hat{\Sigma }{\hat{w}}^{*}}{{\hat{w^{*}}}^{⊤}\Sigma {\hat{w}}^{*}}. \end{array}$$

This is because a variance-minimizing optimization tends to place excess weight on securities whose variances and correlations with other securities are underforecast. In the words of Richard Michaud (44), mean–variance optimizers are “estimation error maximizers.” Bianchi et al. (45) use the VFR to assess risk underforecasting in optimized portfolios. By considering the additional metrics described next, we are able to gauge the accuracy of optimized portfolios themselves, not merely the accuracy of their risk forecasts.

Unlike the VFR, the true variance ratio (TVR) makes sense only for optimized combinations of random variables. TVR is the quotient of the true variance of the true optimum by the true variance of the estimated optimum, and it measures excess variance in the latter:

[27] $$\begin{array}{c} \mathrm{TVR}\left({\hat{w}}^{*}\right)=\frac{w^{*}\Sigma w^{*}}{{\hat{w^{*}}}^{⊤}\Sigma {\hat{w}}^{*}}. \end{array}$$

A more direct measure of the accuracy of an optimized quantity is tracking error, which we define as:

[28] $$\begin{array}{c} {\mathrm{TE}}^{2}\left({\hat{w}}^{*}\right)={\left({\hat{w}}^{*}-w^{*}\right)}^{⊤}\Sigma \left({\hat{w}}^{*}-w^{*}\right), \end{array}$$

for the minimum variance portfolio. Tracking error is widely used by portfolio managers to measure the width of the distribution of the difference in return of two portfolios, and it is commonly applied to measure the distance between a portfolio and its benchmark.

Since these performance metrics require knowledge of the true covariance matrix *Σ*, they cannot be used directly in an empirical study. However, the denominator of VFR, the true variance of the optimized quantity, can be approximated in out-of-sample empirical tests.

### Factor Models, Eigenvalues, and Eigenvectors.

When *p* >  *n*, the sample covariance matrix *S* is singular and so is not a candidate for $\hat{\Sigma }$. Factor models are used throughout the financial services industry and the academic literature to generate full-rank estimates of security return covariance matrices. In the discussion below, we rely on the one-factor model specified in Eq. **10**. Similar results are obtained numerically in the case of multiple factors and nonhomogeneous specific risk in (4) and are supported by theoretical work currently in development.

Writing the factor loadings *β* as a product |*β*|*b* of a scale factor and a unit vector, the population covariance matrix Eq. **11** takes the form

[29] $$\begin{array}{c} \Sigma =(\sigma^{2}{\left|\beta \right|}^{2})bb^{⊤}+\delta^{2}I. \end{array}$$

The quantities *σ*^2^ and |*β*|^2^ are not identifiable from data, but their product *η*^2^ = *σ*^2^|*β*|^2^ is. Thus, we specify an estimator $\hat{\Sigma }$ in terms of an estimator $\hat{b}\in R^{p}$ of unit length and positive estimators ${\hat{\eta }}^{2},{\hat{\delta }}^{2}\in R$ so that

[30] $$\begin{array}{c} \hat{\Sigma }={\hat{\eta }}^{2}\hat{b}{\hat{b}}^{⊤}+{\hat{\delta }}^{2}I. \end{array}$$

In what follows, we use guidance from the HL regime to estimate the identifiable but unobservable quantities *η*^2^ and *δ*^2^ from a data set.

We assume, without loss of generality, that the sample covariance matrix *S* has rank *n*. The leading eigenvalue is denoted *λ*^2^ as before, and we set ℓ^2^ to be the average of the remaining nonzero eigenvalues,

[31] $$\begin{array}{c} \ell^{2}=\frac{tr\left(S\right)-\lambda^{2}}{n-1}, \end{array}$$

where *tr* denotes trace. Under the assumptions of *Theorem 1*, Lemma A.2 of (10) provides the asymptotic relationships between eigenvalues of *S* and factor model parameters. If *p* is sufficiently large,

[32] $$\begin{array}{c} \lambda^{2}\approx \frac{{\left|\beta \right|}^{2}{\left|f\right|}^{2}}{n}+\frac{p}{n}\delta^{2}, \end{array}$$

where *f* = (*f*_1_, *f*_2_…,*f*_*n*_) is the vector of realizations of the common factor return, and

[33] $$\begin{array}{c} \ell^{2}\approx \frac{p}{n}\delta^{2}, \end{array}$$

where ≈ means equality after division by *p*, in the limit as *p* → ∞. An immediate consequence is an approximate expression for the trace of *S* in terms of the elements of the factor model:

[34] $$\begin{array}{c} tr\left(S\right)\approx \frac{{\left|\beta \right|}^{2}{\left|\;f\right|}^{2}}{n}+p\delta^{2}. \end{array}$$

Although we do not have access to | *f*|^2^/*n*, it is an unbiased estimator of the true factor variance *σ*^2^. Relabelling | *f*|^2^/*n* by ${\hat{\sigma }}^{2}$ and applying formulas **32** and **33** gives us estimators:

[35] $$\begin{array}{cc} {\hat{\eta }}^{2} & ={\hat{\sigma }}^{2}{\left|\beta \right|}^{2}\;\approx \;\lambda^{2}-\ell^{2}. \end{array}$$

[36] $$\begin{array}{cc} {\hat{\delta }}^{2} & =\left(n/p\right)\ell^{2}, \end{array}$$

that determine, for any choice of eigenvector estimator $\hat{b}$, the covariance estimator

[37] $$\begin{array}{c} \hat{\Sigma }\left(\hat{b}\right)=\left(\lambda^{2}-\ell^{2}\right)\hat{b}{\hat{b}}^{⊤}+\left(n/p\right)\ell^{2}I, \end{array}$$

with leading eigenvalue *λ*^2^ − ℓ^2^ + (*n*/*p*)ℓ^2^ and trace *λ*^2^ + (*n* − 1)ℓ^2^. The leading sample eigenvalue is approximately equal to the leading population eigenvalue *σ*^2^|*β*|^2^ + *δ*^2^. It also agrees, for *p* >  > *n*, with the S-POET leading eigenvalue estimate of Wang and Fan (19), developed in a regime that includes our spiked model in the HL setting.

The leading population eigenvector *b* remains to be estimated. To help quantify the effect of estimation error on our performance metrics, we use the following two quantities defined for any nonzero eigenvector estimate $\hat{b}$ of *b*. The “optimization bias” $E(\hat{b})$, introduced in (10), is

[38] $$\begin{array}{c} E^{2}\left(\hat{b}\right)=\frac{\left(b,q\right)-\left(b,\hat{b}\right)\left(\hat{b},q\right)}{1-{\left(\hat{b},q\right)}^{2}}. \end{array}$$

and the “eigenvector bias” $D(\hat{b})$, introduced in (11), is

[39] $$\begin{array}{c} D\left(\hat{b}\right)=\frac{{\left(\hat{b},q\right)}^{2}\left(1-{\left(\hat{b},b\right)}^{2}\right)}{\left(1-{\left(\hat{b},q\right)}^{2}\right)\left(1-{\left(b,q\right)}^{2}\right)}, \end{array}$$

where *q* is the unit vector $1/\sqrt{p}$ and ( ⋅ ,  ⋅ ) denotes the Euclidean inner product. Note $E^{2}\left(b\right)=0$, meaning the population eigenvector has zero bias, as desired.

As shown in (10) and (11), and discussed below, these bias measures are substantial contributors to the optimization-based performance metrics VFR, TVR, and TE. A lesson from (10) is that eigenvalue estimates can be less important, for the purpose of optimization in the HL regime, than estimating the leading eigenvector. This is especially true when considering the true variance ${\left({\hat{w}}^{*}\right)}^{⊤}\Sigma {\hat{w}}^{*}$ of an estimated minimum risk portfolio ${\hat{w}}^{*}$ defined by Eq. **25** using the estimated covariance matrix.

### Correcting the Optimization Bias.

In a factor model in the HL regime, JSE can correct the optimization bias, **Eq. 38**, leading to greater accuracy in optimized quantities.

Theoretical guarantees of this assertion are expressed in terms of *η*^2^ = *σ*^2^|*β*|^2^, *δ*^2^, *b*, and their estimates ${\hat{\eta }}^{2}$, ${\hat{\delta }}^{2}$, and $\hat{b}$ from Eq. **30**.

As a consequence of our assumptions on *β*, *η*^2^ is of order *p* asymptotically, so the covariance matrix of data generated by our factor model is spiked. As in the setting of *Theorem 1*, we assume the nondegeneracy condition that |*β*|^2^/*p* tends to a finite positive limit as *p* → ∞.

Theorem 4 (10) and (11). Assume that the angle ∠(*β*, **1**) tends to a limit strictly between zero and *π*/2. Assume that the population covariance matrix is given by Eq. **29**.

1. Asymptotically, the true variance of the estimated minimum variance portfolio is
  [40] $$\begin{array}{c} {\left({\hat{w}}^{*}\right)}^{⊤}\Sigma {\hat{w}}^{*}=\left(\eta^{2}/p\right)E^{2}\left(\hat{b}\right)+o\left(p\right). \end{array}$$
  In particular, the true variance of the estimated minimum variance portfolio is asymptotically independent of eigenvalue estimates but depends only on the eigenvector estimate $\hat{b}$ and the true covariance matrix *Σ*.
2. $\lim_{p\to \infty }E\left(h^{\;\;\text{JSE}}\right)=0$ and $\lim_{p\to \infty }E\left(h\right)>0$ almost surely, where *h* is the leading eigenvector of *S*.
3. Asymptotically, the tracking error of the estimated minimum variance portfolio ${\hat{w}}^{*}$ is
  [41] $$\begin{array}{c} {\mathrm{TE}}^{2}\left(\hat{w}\right)=\frac{\eta^{2}}{p}E^{2}\left(\hat{b}\right)+\frac{\delta^{2}}{p}D\left(\hat{b}\right)+\frac{C}{p}E\left(\hat{b}\right)+o\left(p\right), \end{array}$$
  where *C* is a constant depending on the population covariance matrix, the data, ${\hat{\eta }}^{2}$, and ${\hat{\delta }}^{2}$, but not on $\hat{b}$ (see (11)).

If we denote by *w*_ PCA_ the minimum variance portfolio constructed using the sample eigenvector *h* in Eq. **37**, and *w*_ JSE_ using *h*^ JSE^, parts 1 and 2 of *Theorem 4* imply that TVR(*w*_ PCA_) tends to zero as the dimension *p* tends to infinity, but TVR(*w*_ JSE_) does not. From parts 2 and 3, it follows that TE^2^(*w*_ PCA_) is bounded below, and TE^2^(*w*_ JSE_) tends to zero.

Simulations calibrated to financial markets in refs. (4, 10), and (11) illustrate that these asymptotic properties are already present for values of *p* and *n* that are typical in financial markets. In addition, we observe that the variance forecast ratio is drastically improved by the JSE estimator.

### Numerical Illustration.

Consider the problem of estimating a covariance matrix with a year’s worth of daily observations for stocks in an index like the S&P 500. The observation frequency and size of the data window are limited by empirical considerations: stocks enter and exit the index, markets undergo changes in volatility, and intraday sampling magnifies serial correlation.

In the case at hand, we have approximately *n* = 252 daily observations to estimate a covariance matrix for approximately *p* = 500 variables. Since *p* >  *n*, this problem falls outside the realm of classical statistics. Whether it falls under the HH or HL regime and which performance metrics should be used depend on application details. The example described here illustrates a realistic context in which substantial performance improvements can be achieved using results from the HL regime to correct the leading eigenvector, while corrections of the leading eigenvalue have little value.

We examine a hypothetical market driven by the one-factor model, Eq. **10** with covariance matrix, Eq. **29**. Because the diagonal elements of *S* are unbiased estimators of the population variances, the trace *tr*(*S*) is an unbiased estimator of the sum *tr*(*Σ*) of the population variances. As a consequence, we preserve *tr*(*S*) in our covariance matrix estimators.

We consider the following three data-driven, trace-preserving estimators:

[42] $$\begin{array}{cc} \Sigma_{\;\mathrm{raw}} & =\left(\lambda^{2}-\frac{n-1}{p-1}\ell^{2}\right)hh^{⊤}+\frac{n-1}{p-1}\ell^{2}I, \end{array}$$

[43] $$\begin{array}{cc} \Sigma_{\;\mathrm{PCA}} & =\left(\lambda^{2}-\ell^{2}\right)hh^{⊤}+\left(n/p\right)\ell^{2}I, \end{array}$$

[44] $$\begin{array}{cc} \Sigma_{\;\mathrm{JSE}} & =\left(\lambda^{2}-\ell^{2}\right)\frac{h^{\;\mathrm{JSE}}{\left(h^{\;\mathrm{JSE}}\right)}^{⊤}}{|h^{\;\mathrm{JSE}}{|}^{2}}+\left(n/p\right)\ell^{2}I. \end{array}$$

Here, *Σ*_ raw_ matches the leading eigenvalue and eigenvector of *S* without correction. *Σ*_ PCA_ has the corrected leading eigenvalue but still uses the leading eigenvector *h* to estimate *b*; *Σ*_ JSE_ improves further by substituting *h*^ JSE^ of **Eq. 6** for *h*.

Our factor model parameters are taken approximately from (4) and (10), which contain detailed information about calibration to financial markets. We draw factor and specific returns *f* and *ϵ* independently with mean 0 and standard deviations 16% and 60%, respectively. In the simulation, factor returns are normal, and specific returns are drawn from a *t*-distribution with 5 degrees of freedom. We use this fat-tailed *t*-distribution to illustrate that the results do not require Gaussian assumptions; repeating the experiment with several different distributions including the normal gives similar results.

The factor loadings *β* are inspired by market betas. We draw entries of *β* independently from a normal distribution with mean 1 and variance 0.25 and hold them fixed across time and simulations.

**Fig. 3.**
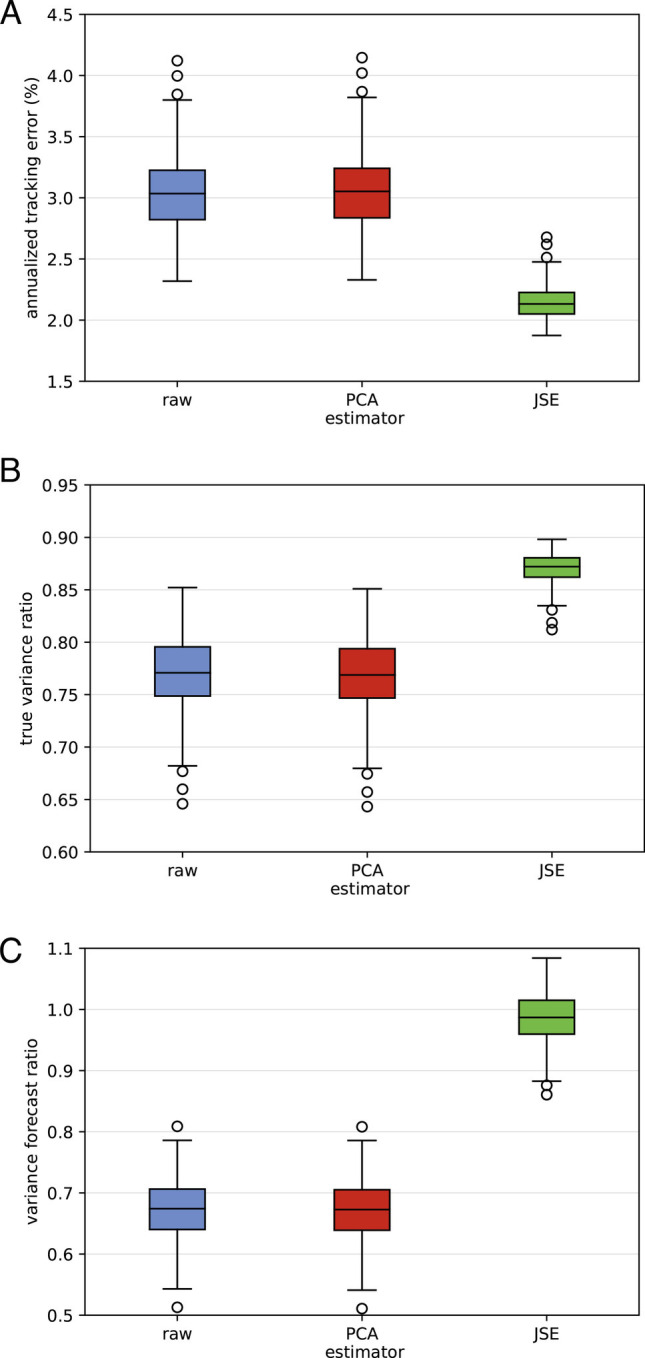
Portfolio-level accuracy metrics for simulated minimum variance portfolios optimized with *Σ*_ raw_, *Σ*_ PCA_, and *Σ*_ JSE_: (*A*) annualized tracking error, (*B*) variance forecast ratio, and (*C*) true variance ratio. A perfect tracking error is equal to zero, and perfect variance forecast ratios and true variance ratios are equal to one. The estimated covariance matrix is based on *n* = 252 observations of *p* = 500 securities. Each boxplot summarizes 400 simulations. The experiments show that eigenvalue correction (PCA) makes no improvement, but the eigenvector correction (JSE) is substantial.

We compare the effect of eigenvalue vs. eigenvector correction on our portfolio performance metrics. In the experiment summarized in Fig. 3, we fix *p* = 500, *n* = 252, and examine the tracking error, variance forecast ratio, and true variance ratio for each of the three estimators *Σ*_ raw_, *Σ*_ PCA_, and *Σ*_ JSE_, with box plots summarizing the values for 400 simulations.

Correcting the leading eigenvalue, from *λ*^2^ to the asymptotically correct *λ*^2^ − (1 − *n*/*p*)ℓ^2^, has little effect compared to the JSE eigenvector correction. Related experiments described in (4) and (10) confirm that improving the accuracy of optimized quantities has negligible dependence on the eigenvalue estimator and relies almost entirely on the choice of eigenvector. All else equal, the magnitude of the improvement in accuracy increases as the dispersion of beta decreases.

Comparing our experiment to the numerical study in (19) illustrates a conclusion from (22): The choice of performance metric materially affects the optimal covariance matrix estimator.

## Summary and Outlook

This article concerns James–Stein for eigenvectors, a shrinkage method that is structurally identical to classical James–Stein. JSE has asymptotic guarantees to improve optimization-based performance metrics in the high-dimension low sample size HL regime. In the context of an empirically motivated one-factor model with a spiked covariance matrix, we show theoretically and illustrate numerically that optimization error is materially reduced by the JSE estimator, while relatively unaffected by eigenvalue correction.

Next steps are to extend the theoretical results to multifactor models and further develop the link between constrained optimization and eigenvector bias. Open problems include an empirical Bayes formulation of JSE for finite *p* and *n* and a more comprehensive understanding of the relationship between performance metrics and errors in eigenvectors and eigenvalues. The notion of “three regimes” is a simplified framework that allows us to organize results, but, in reality, the three regimes belong to a family of largely uninvestigated possibilities. Applications of JSE to GWAS studies, machine learning, and other high-dimension low sample size empirical problems await exploration.

## Data Availability

Python simulation code used to create the boxplots in Fig. 3 and the data in Table 1 is available at https://github.com/kercheval-a/JSE.
